# Point-of-Care Ultrasound in Airway Management

**DOI:** 10.3390/jcm15072726

**Published:** 2026-04-03

**Authors:** Daniele Salvatore Paternò, Luigi La Via, Emilia Lo Giudice, Mario Lentini, Antonino Maniaci, Antoinette Marie Bonaccorso, Rossella Moltisanti, Antonio Putaggio, Federico Pappalardo, Massimiliano Sorbello

**Affiliations:** 1 Department of Anaesthesia and Intensive Care, “Giovanni Paolo II” Hospital, 97100 Ragusa, Italy; paternomd@icloud.com (D.S.P.); rossella.moltisanti@asp.rg.it (R.M.); antonio.putaggio@asp.rg.it (A.P.); 2 Department of General Surgery and Medical-Surgical Specialties, University of Catania, 95123 Catania, Italy; luigilavia7@gmail.com; 3 Department of Anaesthesia and Intensive Care, “Umberto I” Hospital, 94100 Enna, Italy; emilia.logiudice@unikore.it; 4 School of Anaesthesia and Intensive Care, “Kore” University, 94100 Enna, Italy; federico.pappalardo@unikore.it; 5 Department of Ear, Nose and Throat Surgery, “Maggiore” Hospital, 97015 Modica, Italy; mario.lentini@unikore.it (M.L.); antonino.maniaci@unikore.it (A.M.); 6 School of Ear, Nose and Throat Surgery, “Kore” University, 94100 Enna, Italy; 7 Department of General Surgery, “Giovanni Paolo II” Hospital, 97100 Ragusa, Italy; antoinettemarie.bonaccorso@unikore.it; 8 Department of Anaesthesia and Intensive Care, “Morgagni” Clinic, 95123 Catania, Italy

**Keywords:** airway management, ultrasound, POCUS, difficult airway, cricothyrotomy, artificial intelligence

## Abstract

**Background**: Unanticipated difficult airways remain a leading cause of anesthesia-related morbidity and mortality, with traditional bedside predictors demonstrating limited sensitivity. Point-of-Care Ultrasound (POCUS) has emerged as a non-invasive adjunct offering real-time visualization and quantitative measurement of airway anatomy. This narrative review, structured according to the Scale for the Assessment of Narrative Review Articles (SANRA), synthesizes current evidence on POCUS as an adjunct for airway evaluation. We explore the sonoanatomy of the upper airway, the utility of ultrasound in predicting difficult laryngoscopy and intubation, its critical role in emergency front-of-neck access, and the verification of endotracheal tube placement. Furthermore, we discuss the integration of Artificial Intelligence (AI) in image interpretation and the necessity of standardized training curricula. **Methods**: We systematically searched PubMed/MEDLINE, Scopus, and Web of Science for English-language peer-reviewed studies addressing sonographic airway assessment, including sonoanatomy, prediction of difficult laryngoscopy/intubation, guidance for emergency FONA and endotracheal tube confirmation. **Results**: POCUS enhances visualization of critical anatomical structures, may improve anatomical assessment and risk stratification when combined with clinical assessment, and it may provide real-time guidance during emergency procedures. Integration of AI has shown promising diagnostic performance, primarily based on surrogate outcomes. **Conclusions**: Airway ultrasound may represent a shift toward personalized, safer airway management. However, standardized training protocols and validation in diverse clinical settings remain essential. Future research should focus on developing evidence-based algorithms integrating POCUS into airway management guidelines.

## 1. Introduction

Airway management remains a critical task in anesthesia, critical care, and emergency medicine. While most endotracheal intubations proceed uneventfully, the occurrence of an unanticipated difficult airway can result in life-threatening incidents and serious complications [1], including hypoxemia, hemodynamic collapse, or even death, particularly in fragile and critically ill patients [2]. Despite advances in airway devices and techniques, difficult airway-related morbidity persists as a leading cause of anesthesia-related adverse events, underscoring the paramount importance of accurate preprocedural risk assessment.

Airway management planning may reduce complications through careful and meticulous preoperative assessment [3]. Traditional bedside predictors (e.g., the Mallampati score, thyromental distance, neck extension, jaw mobility) have long served as the initial screening tools, yet their diagnostic performance is modest, with pooled sensitivity values typically ranging from 38% to 52% [4]. Consequently, a meaningful proportion of difficult intubations remain unpredicted. Difficult airway management derives from the combination of different anatomical, physiological, environmental, and team-related factors, resulting in the fact that each difficult airway exhibits a specific individual pattern [5]. From this perspective, the use of point-of-care ultrasound (POCUS) as a tool for airway evaluation has emerged as a promising adjunct to standard clinical assessment, given its potential to provide precise and quantitative measurements of anatomical parameters associated with difficult airways [6].

In recent years, POCUS has been increasingly deployed at the bedside for a variety of procedural and diagnostic applications. Its appeal in airway management arises from its non-invasive nature, ability to visualize soft-tissue structures in real-time, and capacity to provide quantitative measurements of anatomical parameters relevant to intubation difficulty [7]. Sonographic measurements such as the distance from skin to epiglottis (DSE), skin to vocal-cord depth, thickness of anterior neck soft tissues, hyomental distance (HMD), and related ratios have been investigated as objective correlates of difficult laryngoscopy and intubation [8,9].

Applications of ultrasound in airway management have been recently extended [10], including assistance in locating the cricothyroid membrane (CTM) for emergency front-of-neck access (FONA), two-point confirmation of correct endotracheal intubation (laryngeal/esophageal findings and symmetric lung sliding) [11], assessment of supraglottic airway device positioning [12], and gastric content and fasting status evaluation [13]. These diverse applications reflect the versatility of ultrasound as a comprehensive airway assessment tool across the peri-intubation continuum.

Despite this momentum, the incorporation of airway ultrasound into routine practice remains variable. While recent meta-analyses have synthesized diagnostic accuracy data for individual ultrasound parameters [8,9], gaps remain in addressing measurement standardization, emergency applications, algorithm integration, and expanding post-intubation roles. Moreover, while many investigations explore preintubation assessment in elective settings, fewer have addressed emergency scenarios or anatomical variations (e.g., morbid obesity, cervical pathology, head and neck masses). The literature thus presents promising but not yet definitive evidence for ultrasound-guided airway assessment [14]. It is worth emphasizing that even with the best-performing clinical screening tools, the prevalence of unanticipated difficult airways remains substantial [15], highlighting the urgent need for adjunctive methods that may improve preintubation preparedness and patient safety.

In the evolving landscape of airway management, the technological proliferation of handheld ultrasound probes and their accessibility at the bedside are critically enabling factors. As the concept of ultrasound as a “fifth pillar” of the physical examination (alongside inspection, palpation, percussion, and auscultation) gains acceptance, the role of ultrasound in airway assessment becomes increasingly viable [16]. This transition from subjective clinical evaluation to objective, image-guided risk stratification enables quantifiable anatomical measurements that show promise in multiple validation studies [8,9], though methodological heterogeneity and limited external validation warrant cautious clinical interpretation.

First, we aim to provide an updated and critical synthesis of the evidence on ultrasound-guided prediction of difficult intubation and ultrasound-based identification of the CTM. Second, we will reflect on how these techniques could be integrated into clinical workflows—highlighting practical considerations, training implications, limitations, and future research directions. Specifically, this review aims to: (1) synthesize current evidence on ultrasound-based prediction of difficult intubation; (2) evaluate the role of ultrasound in emergency FONA; (3) critically appraise limitations including operator dependency and lack of standardization; (4) explore emerging technologies such as artificial intelligence in image interpretation; and (5) propose training frameworks for clinical implementation.

## 2. Materials and Methods

This narrative review was developed in accordance with the Scale for the Assessment of Narrative Review Articles (SANRA) guidelines [17].

### 2.1. Literature Search Strategy and Inclusion Criteria

A structured, comprehensive search of the literature was performed across three major databases: PubMed/MEDLINE, Scopus, and Web of Science. The literature search was conducted on 1 December 2025, covering publications up to 30 November 2025. The search strategy employed a combination of controlled vocabulary (MeSH terms in PubMed) and free-text keywords adapted for each database. Search strings included the following terms: (“airway management” [MeSH] OR “airway ultrasound” [tiab]) AND (“difficult intubation” [MeSH] OR “laryngoscopy” [tiab] OR “cricothyroid membrane” [tiab]) AND (“ultrasonography” [MeSH] OR “point-of-care” [tiab]).

The initial search yielded 1724 records across all databases. After removal of 312 duplicates, 1412 titles and abstracts were screened independently by two reviewers (D.S.P. and L.L.V.) for relevance. Only peer-reviewed English-language articles were considered. Eligible publications included clinical trials, prospective or retrospective cohort studies, diagnostic accuracy studies, meta-analyses, and narrative or systematic reviews that addressed the use of POCUS in airway assessment or FONA. Case reports, editorials without primary data, non-English manuscripts, and conference abstracts without full text were excluded. Disagreements were resolved through discussion, with arbitration by a third reviewer (M.S.) when consensus could not be reached. Fifty-five full-text articles were retrieved and assessed against eligibility criteria. Of these, 15 articles met inclusion criteria and were included in the final qualitative synthesis. The most common reasons for exclusion were: non-English language, lack of primary data on airway ultrasound, conference abstracts without full text, and studies outside the scope of airway assessment.

A PRISMA-style flow diagram was not generated given the narrative nature of this review; however, the selection process adhered to systematic principles of transparency and reproducibility, as recommended by SANRA guidelines.

### 2.2. Selection and Synthesis Approach

After de-duplication, all titles and abstracts were screened independently by two reviewers to assess their relevance to airway POCUS. Full texts of potentially eligible studies were retrieved and assessed for inclusion. Data were extracted concerning study design, patient population, POCUS methodology (probe type, scanning planes, measurement variables), main outcomes, and reported accuracy metrics (sensitivity, specificity, AUC). Given the narrative nature of the review, no formal quantitative synthesis or meta-analysis was conducted.

Instead, a qualitative synthesis was performed, highlighting recurring findings, methodological consistencies and discrepancies, and the evolution of evidence across different clinical contexts (elective, emergent, obese or anatomically distorted airways). Where available, comparative results between POCUS and conventional predictive methods were summarized to contextualize diagnostic performance. Emphasis was placed on reproducibility, operator dependency, and translational potential into routine airway evaluation.

### 2.3. Evidence Level and Bias Considerations

Following SANRA principles [17], we critically appraised the methodological strength and potential bias of included studies. Most available data derive from single-center observational or diagnostic accuracy designs, often with limited sample sizes and heterogeneous definitions of “difficult intubation.” Few studies employed blinded assessment or standardized sonographic protocols, introducing potential selection and observer bias. The absence of uniform measurement thresholds (e.g., for skin-to-epiglottis distance) complicates inter-study comparability.

To mitigate these limitations, findings were discussed in relation to study quality and context rather than pooled. Where systematic reviews or meta-analyses were available, their conclusions were integrated to strengthen the synthesis. Throughout the manuscript, claims were proportionate to the robustness of evidence, and potential confounders—such as operator experience, patient body-mass index, and airway pathology—were explicitly considered.

Finally, this review adhered to SANRA recommendations for transparency and structure, ensuring clear articulation of aims, justification of topic relevance, balanced interpretation of findings, and comprehensive referencing of current literature. The purpose of this methodological rigor is to provide a credible, educationally valuable synthesis that may guide both clinical practice and future research design. Given the narrative scope and the absence of quantitative synthesis, we did not conduct formal risk-of-bias assessment using tools such as QUADAS-2 or Cochrane ROB; however, study quality was critically appraised descriptively, and findings were interpreted in the context of methodological rigor and potential sources of bias.

## 3. Ultrasonographic Anatomy of the Upper Airway

**Figure 1:**
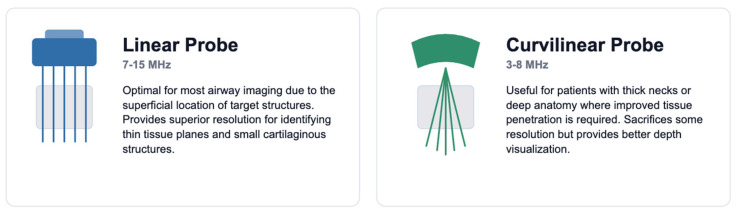
Ultrasound transducers for airway assessment. Linear array probe (7–15 MHz, **left**) provides superior resolution for superficial structures (epiglottis, vocal cords, cricothyroid membrane); curvilinear probe (3–8 MHz, **right**) enables deeper penetration for tongue base and hyoid bone visualization.

A systematic and reproducible knowledge of the anatomical relationships between the tongue, epiglottis, hyoid bone, laryngeal cartilages, vocal cords, and trachea enables clinicians to identify critical landmarks for intubation, ventilation, and emergency FONA. Over the past decade, high-frequency POCUS has become increasingly commonly used to delineate these structures in both normal and difficult airways, allowing real-time assessment of dynamic changes during respiration and airway manipulation [18] (Figure 1).

### 3.1. Tongue and Floor of the Mouth

**Figure 2:**
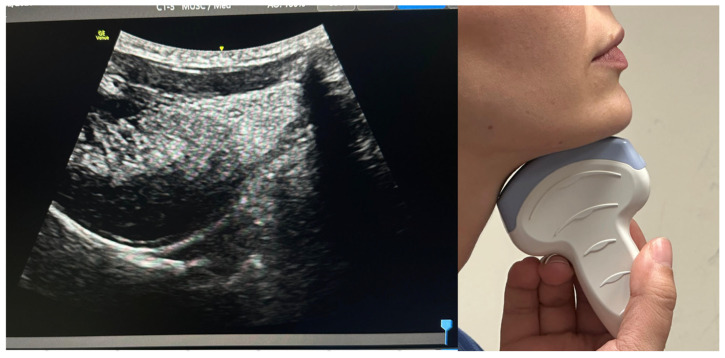
Submental sagittal ultrasound view (curvilinear probe, 3–5 MHz).

The tongue is best visualized using a curvilinear probe placed in the submental region with the patient in the supine position and mouth closed. In the sagittal (midline) plane, the geniohyoid and mylohyoid muscles appear as paired hypoechoic bands separated by a hyperechoic midline raphe. The intrinsic musculature of the tongue forms a heterogeneous echotexture anterior to the hyoid bone (Figure 2). Measurements such as the HMD and the hyomental distance ratio (HMDR)—the ratio of HMD in neutral versus extended neck positions—have been proposed as sonographic predictors of difficult laryngoscopy [19,20]. These metrics provide an objective quantification of anterior neck soft-tissue compliance and have demonstrated moderate correlation with Cormack–Lehane laryngoscopic grades.

### 3.2. Epiglottis and Pre-Epiglottic Space

**Figure 3:**
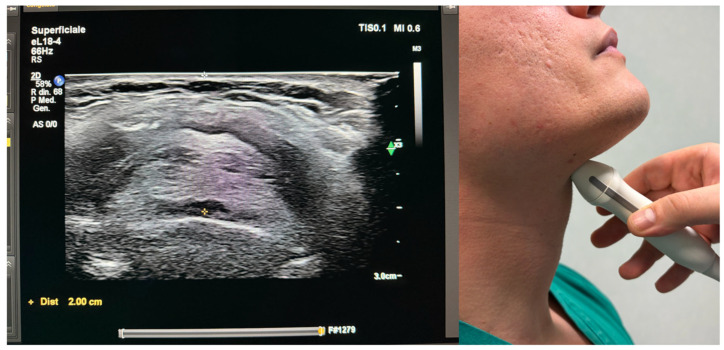
Transverse view at thyrohyoid membrane level (linear probe, 10–12 MHz).

The epiglottis can be imaged by placing the linear probe transversely over the thyrohyoid membrane, just superior to the thyroid cartilage. It appears as a curved hypoechoic structure with a bright posterior border, representing the air–mucosa interface of the epiglottic surface (Figure 3) [21]. The DSE—a parameter measured from the skin surface to the midpoint of the epiglottic shadow—has gained attention as a reliable predictor of difficult laryngoscopy, with thresholds between 2.3 and 2.5 cm showing acceptable diagnostic accuracy [22].

The pre-epiglottic space (PES), filled with adipose tissue, appears hypoechoic, and its thickness may influence epiglottic mobility during laryngoscopy. Evaluating the PES also assists in differentiating normal from pathological conditions, such as epiglottic edema or neoplastic infiltration, which can affect intubation safety.

### 3.3. Hyoid Bone and Laryngeal Cartilages

The hyoid bone is identified as a bright, curved echogenic line casting a posterior acoustic shadow. Its visualization is essential for orienting the examiner during longitudinal and transverse scans, acting as a landmark that separates the suprahyoid and infrahyoid compartments [23]. The thyroid cartilage appears as a paired hypoechoic structure with an intervening midline notch, while the cricoid cartilage is more circular and produces a stronger posterior acoustic shadow due to its complete ring morphology [24]. With age, ossification of these cartilages increases, altering echogenicity and sometimes complicating image interpretation. Nevertheless, recognition of the laryngeal framework provides guidance for identifying the CTM and tracheal rings. Precise elective localization of the CTM using this bony framework may reduce misidentification rates during emergency FONA, particularly in obese or edematous patients [24].

### 3.4. Vocal Cords and Glottic Structures

**Figure 4:**
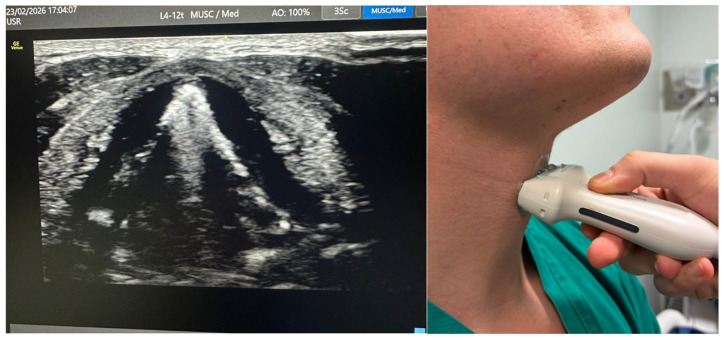
Transverse view of true vocal cords (linear probe, 10–12 MHz, thyroid cartilage level). True vocal cords appear as paired hypoechoic triangular structures forming a ‘seagull sign’ during phonation.

Sonographic evaluation of the vocal cords can be achieved using either a transverse scan through the thyroid cartilage (Figure 4) or a longitudinal midline approach through the CTM. The true cords appear as paired hypoechoic linear structures that move symmetrically during phonation, while the false cords demonstrate less mobility and greater echogenicity [25]. Dynamic POCUS allows visualization of cord motion, which is useful in assessing recurrent laryngeal nerve palsy or verifying endotracheal tube placement. The air–mucosa interface beneath the cords produces a distinct hyperechoic line, aiding identification even when direct visualization is difficult [26]. Dynamic assessment during phonation enables detection of vocal cord pathology that may complicate intubation, while post-intubation scanning confirms symmetric cord motion and absence of arytenoid dislocation.

### 3.5. Trachea and Subglottic Region

**Figure 5:**
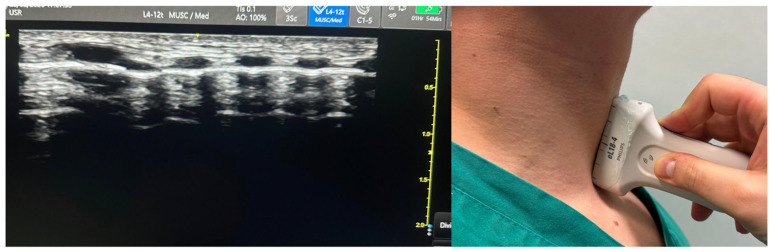
Longitudinal midline tracheal view (linear probe, 7–10 MHz). Tracheal cartilaginous rings appear as hypoechoic curvilinear bands (‘string of pearls’) with posterior reverberation artifact.

Below the CTM, the trachea appears as a series of hypoechoic cartilaginous rings, often referred to as a “string of pearls”, surrounding a central air column that generates posterior reverberation artifacts (Figure 5). The tracheal midline is verified by symmetric visualization of the lateral lobes of the thyroid gland, an important step during preintubation airway assessment and percutaneous tracheostomy planning, with special emphasis in head and neck surgery [27]. POCUS may also help identifying abnormal tracheal anatomy or pathologic status of tracheal cartilaginous rings [28]. The distance from skin to anterior tracheal wall can be measured to anticipate challenges in surgical or needle cricothyrotomy, particularly in obese patients or those with cervical swelling [29].

## 4. Sonographic Predictors of Difficult Intubation

The quest to reliably predict a difficult intubation remains one of the most persistent challenges in anesthesiology and airway management. Traditional bedside tests—such as the Mallampati classification, thyromental distance, and upper lip bite test—suffer from limited sensitivity and specificity, with many difficult airways occurring unexpectedly despite apparently favourable assessments [3,30,31,32]. In recent years, POCUS has emerged as a powerful, non-invasive adjunct capable of quantifying airway anatomy and tissue compliance in real time. Sonographic evaluation provides both quantitative and qualitative information that can refine preoperative risk stratification and augment conventional clinical predictors.

### 4.1. Quantitative Parameters

#### 4.1.1. Anterior Neck Soft-Tissue Thickness

Anterior neck soft-tissue thickness (ANS) represents one of the most extensively studied quantitative predictors. It is typically measured as the distance from the skin surface to the anterior aspect of the airway structures, such as the hyoid bone, epiglottis, or vocal cords. In a landmark study, Ezri et al. demonstrated that a greater distance between the skin and the epiglottis was strongly correlated with difficult laryngoscopy [33]. The DSE, measured in the mid-sagittal plane at the thyrohyoid membrane, has since become one of the most validated parameters. Thresholds between 2.3 and 2.5 cm have been proposed as predictive cut-offs, with pooled sensitivity and specificity of approximately 75% and 86%, respectively [34]. It is important to note that most DSE validation studies use Cormack–Lehane grade ≥ 3 as the outcome; whether DSE thresholds predict actual intubation failure (vs. poor laryngoscopic view) remains less well-established, particularly when video laryngoscopy is available. However, significant heterogeneity exists in reported DSE cut-offs across studies, ranging from 1.6 cm to 2.75 cm. This variability stems from several sources: methodological differences (transverse vs. sagittal scanning planes, linear vs. curvilinear probes, degree of probe pressure), patient population characteristics (elective surgical patients vs. emergency cases, BMI distribution, ethnic composition), and operator experience. Moreover, some studies measure DSE at the level of the hyoid bone, while others target the thyrohyoid membrane, introducing anatomical inconsistency.

From a clinical implementation perspective, this heterogeneity implies that institutions cannot simply adopt published cut-offs without local validation. Therefore, pragmatic recommendations include: (1) selecting a standardized scanning protocol institution-wide (e.g., mid-sagittal plane, minimal probe pressure, measurement at thyrohyoid membrane); (2) conducting a pilot validation study of 50–100 patients to calibrate cut-offs to local demographics; and (3) integrating POCUS findings with clinical predictors rather than relying on a single sonographic threshold. Until international consensus protocols emerge, context-dependent interpretation remains essential.

The pretracheal depth—defined as the distance between the skin and the anterior tracheal wall at the level of the suprasternal notch—has also been evaluated, particularly in obese patients. Thick pretracheal soft tissue (>28 mm) increases the risk of failed visualization of the vocal cords and difficult intubation [35]. These findings are consistent with the notion that excessive anterior neck adiposity impairs laryngoscopic alignment of oral, pharyngeal, and laryngeal axes.

#### 4.1.2. Hyomental Distance and Ratio

The HMD is the linear measurement between the hyoid bone and the mentum, typically obtained in the midline sagittal view. This distance shortens in patients with anterior larynx positioning or reduced mandibular mobility—both known contributors to intubation difficulty [9]. However, because absolute HMD values vary by sex, height, and ethnicity, the HMDR was introduced as a more standardized metric. An HMDR < 1.2 has been associated with difficult laryngoscopy, reflecting limited cervical extension or soft-tissue compliance [36]. As with DSE, HMDR primarily predicts difficult laryngoscopy rather than intubation failure per se. Moreover, HMDR thresholds exhibit population-specific variability; institutions should validate local cut-offs before clinical implementation [9,36]. These quantitative indices offer an objective alternative to subjective clinical estimates of airway flexibility. Nevertheless, HMDR thresholds also exhibit inter-study variability (ranging from <1.2 to <1.4 depending on patient positioning protocols and neck extension definitions), and its predictive accuracy may be population-specific; for instance, HMDR performs better in patients with limited cervical mobility but shows reduced discriminatory power in younger, flexible cohorts [9,36].

#### 4.1.3. Composite Ultrasound Scores

Recent efforts have explored multivariate POCUS indices combining several parameters (DSE, pretracheal depth, and HMDR). De Luis-Cabezón et al. proposed a composite “Airway US Score” integrating three sonographic predictors with two clinical variables, improving overall predictive accuracy compared with any single measurement [14]. However, this score was validated against laryngoscopic grade rather than intubation success. Furthermore, composite scores require validation across diverse populations and clinical settings (elective vs. emergency, varying BMI distributions) before widespread adoption. Although not yet universally validated, such integrated models illustrate the potential of POCUS-based scoring systems to augment traditional airway algorithms.

### 4.2. Qualitative Markers

Beyond quantitative measures, qualitative sonographic findings provide contextual insight into airway configuration. The visibility of the epiglottis, for example, is a qualitative surrogate for anatomical accessibility during laryngoscopy. In well-visualized airways, the epiglottis appears as a distinct hypoechoic arc with posterior reverberation; in contrast, blurred or absent visualization often correlates with deep-seated or anteriorly displaced laryngeal structures [37]. Similarly, the hyoid–mandible position—reflected in the relative orientation of the hyoid bone to the mandibular symphysis—can signal potential difficulty when the hyoid lies unusually posterior or inferior, indicating a reduced mandibular space [38].

In certain populations, such as patients with obstructive sleep apnea (OSA) or obesity, these qualitative patterns are accentuated by fatty infiltration and redundant soft tissue, further supporting the role of sonography as a dynamic anatomical mapping tool [39]. Moreover, POCUS visualization of the tongue base, vallecula, and epiglottic edge can help identify patients in whom direct laryngoscopy may fail, guiding early use of video-assisted or fiberoptic techniques.

## 5. Comparative Performance and Clinical Integration of Ultrasound-Based Assessment

In the evolving landscape of airway management, the comparative performance of POCUS-based assessment versus traditional clinical screening is a critical area of investigation.

### 5.1. Meta-Analyses and Prospective Trials Comparing POCUS and Clinical Assessments

Several systematic reviews and meta-analyses have evaluated the diagnostic accuracy of POCUS-derived airway parameters compared with conventional bedside tests. For example, one meta-analysis found that POCUS metrics across three domains—anterior tissue thickness, anatomical position, and oral space—yielded a pooled sensitivity of 76% (95% CI 71–81%) and specificity of 77% (95% CI 72–81%) for anterior tissue thickness, and an AUROC of 0.83, whereas the anatomical position domain yielded even higher specificity (86%) and AUROC (0.87) [8]. In general, POCUS-derived measurements outperform individual bedside tests in terms of both sensitivity and specificity. For instance, Anushaprasath et al. reported that DSE had an area under the ROC curve (AUC) of 0.87 compared to 0.68 for the Mallampati score [40]. Similarly, the HMDR demonstrated greater predictive value than thyromental distance alone, highlighting the advantage of dynamic, anatomically grounded measurements.

Nevertheless, combining POCUS with conventional predictors yields the best diagnostic performance. A prospective trial found that integrating sonographic DSE with the modified Mallampati and upper lip bite tests increased the positive predictive value for difficult laryngoscopy from 48% to 86% [8]. Such evidence reinforces the concept that POCUS should complement—not replace—clinical assessment.

Despite these promising results, several caveats remain. First, operator dependency and the absence of standardized measurement protocols introduce variability in reported cut-offs. Second, many studies have small sample sizes or selective inclusion criteria, limiting generalizability. Finally, diagnostic accuracy studies often use Cormack–Lehane grade ≥ 3 as a surrogate for difficult intubation, which may not fully capture real-world airway challenges. Furthermore, population representativeness remains a critical limitation. Larger, multicentre trials with standardized definitions are required to confirm the clinical utility of these parameters [9,41].

Prospective trials have further demonstrated that POCUS measurements are significantly different between patients with easy versus difficult direct laryngoscopy. For instance, one study reported mean differences in DSE of 0.38 cm (95% CI 0.17–0.58), DSVC (distance skin to vocal cords) of 0.18 cm (95% CI 0.01–0.35) and DSHB (distance skin to hyoid bone) of 0.23 cm (95% CI 0.08–0.39) in difficult vs. easy groups [42]. These findings suggest that POCUS may provide a more sensitive anatomical assessment than observation of external landmarks alone (Table 1).

Comparisons with clinical prediction tools consistently show that traditional tests (e.g., Mallampati classification, thyromental distance, upper lip bite test) display high specificity but disappointingly low sensitivity. For example, a large meta-analysis of these clinical methods found sensitivities of around 0.38–0.52 with specificities of approximately 0.83–0.86 [41]. In contrast, POCUS appears to deliver higher sensitivity while retaining similar specificity in many studies, suggesting potential for improved detection of at-risk airways.

### 5.2. Integration into Preoperative Screening

**Figure 6:**
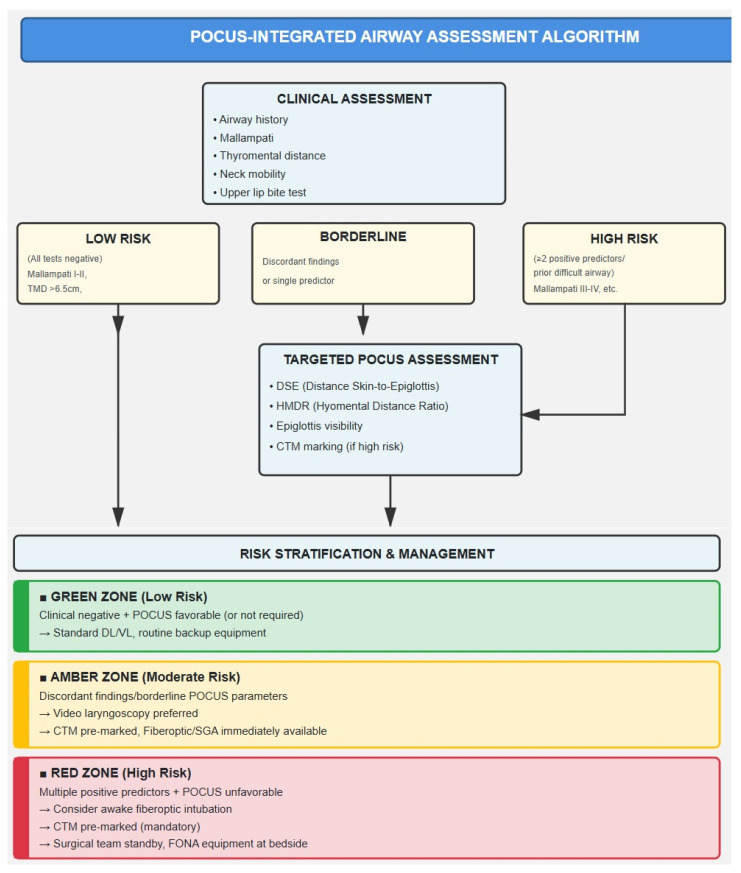
POCUS-integrated airway assessment algorithm.

POCUS may be viewed not as a replacement for clinical evaluation, but as a complementary tool. A practical workflow could involve initial clinical screening (Airway history, Mallampati, thyromental distance, neck mobility, interincisal distance and upper lip bite test), followed by targeted POCUS in patients with equivocal or borderline findings, significant risk factors (obesity, cervical pathology, prior difficult airway) or anatomical uncertainty (Figure 6).

Practical implementation of airway POCUS within clinical workflows requires a structured, multidisciplinary approach. The first step is the development of standardized protocols that clearly define which sonographic parameters—such as the DSE), HMDR, and anterior neck soft-tissue thickness—should be measured, alongside institution-specific cut-offs validated in local populations. Adequate training is equally essential: anesthesiologists and airway teams must acquire the skills to obtain reproducible scans, accurately interpret images, and contextualize findings within each patient’s airway profile. POCUS results should then be incorporated into preoperative airway risk charts, categorizing patients by risk level (for example, green for low risk, amber for moderate risk where video laryngoscopy may be advisable, and red for high risk requiring awake intubation planning). These findings should also be linked to specific clinical decision pathways, triggering the use of advanced airway strategies or ensuring the immediate availability of surgical airway equipment in high-risk cases. Finally, continuous audit and feedback mechanisms are vital—tracking metrics such as first-pass success, incidence of unexpected difficult intubations, and complications—to refine institutional thresholds and ensure ongoing quality improvement.

Lastly, future research must move beyond diagnostic accuracy to evaluate outcome-based endpoints: does adding POCUS reduce unexpected difficult intubations, decrease complications, or improve first-pass success? Without outcome data, widespread adoption remains aspirational.

## 6. POCUS-Guided CTM Identification

POCUS has gained traction for identifying the CTM when anatomical landmarks are obscured by obesity, neck masses, or trauma [29,51]. Multiple studies demonstrate that POCUS outperforms manual palpation, facilitating preparation for emergency cricothyrotomy [52,53].

Accurate CTM identification is critical in “cannot intubate, cannot oxygenate” (CICO) scenarios. Traditional palpation often fails in obese or anatomically distorted patients, contributing to high FONA failure rates. POCUS-guided CTM localization enhances anatomical accuracy and procedural safety in both elective and emergent settings [44,54] and should be considered during preprocedural airway assessment.

### 6.1. Techniques and Scanning Protocols

POCUS approaches to CTM identification employ either transverse (short-axis) or longitudinal (sagittal) planes. In the transverse approach, a high-frequency linear probe is placed at the thyroid cartilage notch and scanned caudally until the hyperechoic air–mucosa interface is visualized, identifying the CTM between thyroid and cricoid cartilages. The longitudinal “string-of-pearls” technique aligns the probe sagittally, tracking cricoid and tracheal rings as hypoechoic beads with the CTM appearing as an echoless gap. A systematic review found the transverse technique to be faster, though both methods showed comparable accuracy [9]. Probe choice and scanning technique should be tailored to patient anatomy. A high-frequency linear transducer (10–15 MHz) is ideal for superficial structures, but obese or thick-necked patients may require a lower-frequency curvilinear probe for deeper penetration. Optimal neck positioning (neutral or slightly extended) is essential to maximize acoustic windows and minimize sonoanatomic distortion.

### 6.2. Accuracy in Normal and Difficult Neck Anatomy

POCUS significantly outperforms palpation for CTM identification across anatomical scenarios. A landmark randomized trial of 223 subjects with difficult neck anatomy found accurate localization within 5 mm of CT reference in 81% of POCUS attempts versus 8% with palpation (*p* < 0.0001) [9]. In obese obstetric patients, POCUS correctly identified the CTM in 71% versus 39% with palpation (*p* = 0.015), though POCUS required slightly more time (23.5 s vs. 16.9 s) and was rated more technically challenging [16]. A systematic review of 14 studies confirmed POCUS superiority for CTM localization—particularly in difficult anatomy—and more reliable objective definition of neck landmarks compared to palpation [44]. It is worth noting that although promising, POCUS identification of the CTM has not yet been definitively shown to improve clinical outcomes (e.g., first-attempt success of cricothyrotomy or reduced complications), as most studies remain observational or cadaveric in nature. The adoption of POCUS for CTM marking, however, is increasingly recommended as part of a preintubation “double-set-up” in anticipated difficult airway cases [4,29].

### 6.3. Role in Emergency Airway Access and Decision-Making

In the algorithm of difficult airway management, especially when emergency FONA is a potential step, preprocedural POCUS marking of the CTM provides several strategic advantages. First, it enables preemptive planning: if the site of cricothyroidotomy is identified and marked before the induction of anaesthesia, the operative window in a CICO crisis is narrowed and anatomical surprises are reduced. Secondly, it enhances procedural confidence: multiple studies report that anesthesiologists who mark the CTM under POCUS are more likely to feel prepared for emergency airway access than those relying on palpation alone. For instance, post-training survey data revealed that 79% of emergency medicine residents reported that focused POCUS training significantly improved their comfort level with CTM identification [55].

However, POCUS is not recommended once a CICO scenario is ongoing and the airway is already lost because the time required to set up the probe and scan may delay airway access. Instead, the technique is best applied in the preinduction phase when there is anticipated airway risk and time for planning [46,47].

Proper technique is essential to ensure accurate sonographic identification of the CTM. A generous amount of gel should be applied to minimize compression artifacts in the anterior neck, and the probe must be placed gently to avoid distortion of surrounding soft tissues. During scanning, the operator should clearly recognize key anatomical landmarks—the thyroid cartilage, appearing as a triangular hyperechoic ridge; the cricoid cartilage, visible as a circumferential hypoechoic ring; and the tracheal rings, forming the characteristic “string-of-pearls” pattern. The CTM is located in the interval between the thyroid and cricoid cartilages. Optimal patient positioning involves a slight neck extension, or a ramped position if necessary, to improve visibility while avoiding excessive extension that may distort POCUS windows. Once the CTM is visualized, pre-marking the site with a sterile skin marker facilitates immediate recognition during airway emergencies. Documentation of the POCUS image and the marked location is recommended for clinical records and teaching purposes. Finally, structured, short-duration training sessions—such as in-house airway POCUS workshops—have been shown to promote rapid acquisition and durable retention of CTM identification skills even among non-radiologists [55].

## 7. Clinical Applications and Training Implications

The incorporation of airway POCUS into everyday clinical practice—spanning anesthesiology, emergency medicine, and critical care—represents a significant shift toward image-guided airway management. At the same time, the successful deployment of these techniques hinges on structured training programs, simulation-based education, and credentialing frameworks to ensure competence, reduce variability, and mitigate operator-dependence [56].

### 7.1. Incorporation in Anesthesiology, Emergency Medicine, and Critical Care Practice

In the perioperative arena, POCUS of the upper airway is increasingly used for preintubation screening for difficult anatomy, confirmation of endotracheal tube placement, localization of the CTM, and dynamic monitoring of airway changes (e.g., tongue base swelling, edema). In emergency departments and critical care units, portable POCUS allows rapid bedside assessment when physical anatomy is distorted. While some authors suggest that airway POCUS may improve first-pass success rates [43], this hypothesis requires validation in prospective outcome studies. Beyond the core applications addressed in this review—difficult airway prediction and CTM identification—POCUS has been explored for esophageal intubation exclusion [57,58,59], supraglottic device positioning [60], secretions assessment [61], lung separation procedures [62], and gastric content evaluation [63]. Experience during the COVID-19 pandemic demonstrated POCUS versatility in airway management [64]. However, detailed discussion of these ancillary applications falls outside this review’s primary scope.

Despite the promise, actual adoption remains inconsistent. A recent survey of anesthesiology residency programs in the U.S. found that only 21% had a formal airway POCUS curriculum, and airway POCUS was ranked lowest among POCUS topics in terms of training emphasis [48]. Barriers included a lack of faculty trained in airway sonography, limited dedicated time for instruction, and absence of standardized competency assessment. Translation into everyday practice requires organizational commitment, equipment availability, and integration into airway algorithms.

### 7.2. Simulation-Based Education and Credentialing Models

Simulation offers a structured, safe, and reproducible environment to teach airway POCUS before real-world patient application. Hybrid airway models, high-fidelity mannequins, and virtual reality platforms allow learners to visualise sonoanatomic landmarks, practice probe manipulation, perform measurements, and interpret images under guided supervision. A recent low-cost hybrid airway model for nursing students showed high feasibility and learner satisfaction in training basic airway sonography skills [65,66,67]. These educational modalities support deliberate practice, feedback, and error correction without patient risk.

Credentialing frameworks and direct assessment of competence are increasingly recognised as vital. The surveyed anesthesiology programs reported relying on combinations of hands-on skills assessment, written exams, image review and minimum scan numbers—but 60% of programs lacked formal competency assessment for airway POCUS [68]. This gap underscores the need for standardised curricula. For example, the American Society of Regional Anesthesia and Pain Medicine (ASRA) expert panel recommended minimum study volumes: 30 airway exams, 30 lung scans, and 20 gastric scans in a broader POCUS curriculum [69,70,71]. While not airway-specific, this provides a useful benchmark.

In addition, embedding POCUS findings into institutional airway protocols (e.g., marking high-risk patients, prompting video laryngoscope or awake intubation) fosters translation into decision-making workflows and promotes sustained use. Combining simulation, credentialing and clinical integration supports the transition from technical skill to cognitive decision-making in airway management.

## 8. Limitations and Controversies

While the adoption of POCUS in airway management promises significant benefits, it is essential to critically examine persistent limitations, emerging controversies, and the future-oriented avenues—such as artificial intelligence (AI) and automated analysis—that may overcome current hurdles.

### 8.1. Operator Variability and Lack of Standardization

One of the most consistently cited weaknesses of airway POCUS is operator dependence. Imaging acquisition, landmark identification, probe pressure, and measurement techniques vary among practitioners, contributing to significant inter-observer and intra-observer variability [49]. For example, one feasibility study in an emergency department setting found inter-rater intraclass correlation coefficients (ICCs) ranging from 0.76 to 0.88 for various upper airway measurements—but only 0.57 for epiglottic thickness, indicating that even in controlled settings, variability remains [50]. Multi-institutional studies confirm that inter-rater reliability improves significantly when standardized measurement protocols and structured reporting templates are employed [72]. However, real-world adoption of such protocols remains limited outside academic centers [73].

Compounding this is the absence of standardized scanning protocols for airway assessment. Despite meta-analyses demonstrating promising values for parameters such as DSE or HMDR [9], heterogeneity among studies in head position, measuring plane, probe frequency, patient population (elective vs. emergency vs. obese) and cut-off values limits generalizability [44]. As one recent systematic review concluded: “high clinical and methodological heterogeneity has been found between studies … it is not currently possible to reach a definitive conclusion before better standardization of POCUS assessment” [9].

The lack of consensus on measurement definitions and thresholds also means that clinical implementation is inconsistent. For instance, DSE cut-offs vary widely (from 1.6 cm to 2.75 cm) across studies, making institution-specific calibration essential prior to routine use [54]. The clinical consequences of this variability are significant. A clinician applying a 2.3 cm DSE threshold derived from a European study to an obese North American cohort may encounter unacceptably high false-negative rates if the local population’s mean DSE differs by even 3–5 mm due to demographic factors. Similarly, failure to standardize probe pressure can artificially compress soft tissues, reducing measured DSE by 2–4 mm and falsely reassuring the operator about airway difficulty [50].

To mitigate these challenges, several centers have adopted standardized reporting templates (analogous to structured radiology reports) specifying: probe type, measurement plane, patient position, anatomical landmarks, and quality assurance checkpoints (e.g., visualizing both hyoid and cricoid cartilages before measuring DSE). Early evidence suggests that such templates improve inter-rater ICC from 0.57 to 0.65 (unstructured) to 0.82–0.88 (structured protocol) [72], though widespread adoption outside research settings remains limited. Therefore, despite promising diagnostic accuracy metrics, routine adoption remains hindered by these standardization gaps.

### 8.2. Technological Challenges (Portable Devices and AI-Based Measurement)

Emerging technology brings new promise but also new challenges. The proliferation of portable and handheld POCUS devices offers broader access, but device-based variability—including probe frequency, image resolution, gel quality, user interface and built-in measurement tools—can influence the accuracy of airway scans. Some studies point out that suboptimal POCUS windows (e.g., in patients with subcutaneous emphysema, thick necks or anterior neck masses) remain a barrier to consistent imaging [7].

The application of AI and machine learning to airway ultrasound represents an emerging but largely experimental field. In lung POCUS, AI guidance has enabled non-experts to acquire diagnostic-quality images with success rates over 98% [74]. Though airway-specific AI work is still nascent, a narrative review of AI in difficult airway assessment concluded that deep-learning models using images plus clinical data offer promising gains but are “limited by data-quality, class imbalance, and generalizability” [30]. A recent article describes deep-learning workflows for airway landmark detection in neck POCUS, noting that synthetic image-generation methods may help remedy class imbalance but clinical deployment remains some distance off [74].

However, critical appraisal reveals substantial limitations: most AI algorithms are trained on small, single-center datasets (<500 images) with limited diversity, and performance metrics frequently overestimate real-world accuracy due to overfitting and lack of external validation. No AI algorithm for airway ultrasound has received regulatory clearance (FDA, CE Mark), and the pathway to clinical implementation requires multi-center validation. Also, current technologies remain in early experimental (automated landmarking, image quality feedback) or mid-stage development (automated measurement algorithms) phases.

### 8.3. External Validity and Population Generalizability

Beyond methodological heterogeneity, a fundamental limitation of the current evidence base is restricted population representation and limited real-world validation. Systematic examination of included studies reveals several consistent exclusion patterns that constrain generalizability:

- Emergency and critically ill populations: Most diagnostic accuracy studies enrolled elective surgical patients (ASA I–III), with <15% of the literature addressing emergency department or ICU intubations [43,48]. Yet these high-acuity settings (where unanticipated difficult airways are most prevalent and consequences most severe) differ fundamentally in terms of: (1) time constraints preventing deliberate POCUS acquisition; (2) physiological instability (hypoxemia, shock) demanding immediate intervention; (3) operator stress and cognitive load; and (4) patient positioning limitations (inability to optimize neck extension or ramping).
- Anatomically complex patients: Individuals with morbid obesity (BMI > 40 kg/m^2^), cervical spine pathology (fractures, degenerative disease, ankylosing spondylitis), head/neck malignancies, post-radiation fibrosis, or airway masses (populations at highest risk for difficult intubation) are systematically underrepresented or excluded from POCUS studies due to poor acoustic windows or ethical concerns about delaying airway management [14,39]. Yet these are precisely the patients who might benefit most from enhanced anatomical delineation.

### 8.4. Surrogate Endpoints and Absence of Patient-Centered Outcome Evidence

Perhaps the most fundamental limitation of the current airway POCUS evidence base is the near-complete absence of patient-centered outcome data. The overwhelming majority of studies employ Cormack–Lehane laryngoscopic grade as the primary outcome, a surrogate endpoint that measures laryngoscopic view, not intubation success. Critical patient-centered endpoints remain unstudied: first-pass intubation success (the most robust predictor of favorable outcomes [2]), airway-related complications (hypoxemia, esophageal intubation, aspiration, cardiovascular collapse), procedural metrics (time, number of attempts, rescue technique requirement), and whether POCUS information changes management decisions in ways that improve outcomes [1,11,15].

The current evidence establishes that POCUS can predict anatomical difficulty more accurately than traditional bedside tests, but diagnostic accuracy does not equal clinical utility [3,47].

## 9. Conclusions

POCUS has emerged as a promising adjunct for airway evaluation, offering real-time visualization of upper airway structures, quantifiable predictive parameters, and accurate localization of the CTM for emergency FONA. Its strongest current value lies in educational applications and preoperative planning for anticipated difficult airways, where time permits standardized image acquisition by trained operators. However, widespread clinical implementation is premature given persistent limitations: operator dependency, lack of standardized measurement protocols, population-specific cut-off variability, and—most critically—absence of outcome-based evidence demonstrating that ultrasound-guided assessment reduces unanticipated difficult intubations, first-pass failure, or patient harm. Future integration into routine practice will require multicenter validation studies with patient-centered endpoints, consensus standardization of techniques, and structured competency-based training curricula before definitive guideline incorporation can be justified.

## Figures and Tables

**Table 1:** Strengths and Limitations of Point-of-Care Ultrasound for Airway Assessment.

Domain	Key Findings	Strength of Evidence/Limitations
Diagnostic performance for difficult laryngoscopy	Meta-analyses report pooled sensitivity 76–86% and specificity 77–86% for predicting Cormack–Lehane grade ≥ 3, with AUROC 0.83–0.87 for DSE and anatomical position parameters [8,9,41]	Critical caveats: (1) Most studies use laryngoscopic grade (surrogate outcome), not intubation success; (2) marked heterogeneity in cut-offs (DSE: 1.6–2.75 cm), scanning protocols, and patient populations limits generalizability; (3) small sample sizes (<200 patients in most trials); (4) limited validation in emergency/critically ill cohorts [9,34,42,43]
Complementary value when combined with clinical assessment	Integration of POCUS with traditional predictors (e.g., DSE + Mallampati + upper lip bite) increases positive predictive value from 48% to 86% in selected studies, suggesting additive benefit [8,14]	Uncertain clinical impact: No randomized trials demonstrate that adding POCUS reduces unanticipated difficult intubations, first-pass failure, or complications. Benefit may be limited to borderline-risk patients where clinical assessment is equivocal [3,15,43]
CTM localization	POCUS outperforms palpation for CTM identification (81% vs. 8% accuracy within 5 mm of CT reference in difficult anatomy; 71% vs. 39% in obese obstetric patients), enabling pre-marking before anticipated difficult airway cases [9,44,45]	Implementation gaps: (1) Benefit demonstrated in elective pre-marking, not during active “cannot intubate, cannot oxygenate” crisis (where time delay is prohibitive); (2) improved anatomical localization has not been shown to reduce cricothyrotomy complications or improve FONA success rates in outcome studies [29,46,47]
Fundamental limitations	Operator dependence: Image acquisition, landmark identification, and measurement accuracy require structured training (learning curve ~30–50 scans for competence). Lack of standardization: No consensus on probe type, scanning planes, patient positioning, or measurement definitions—precludes universal protocols. Population-specific performance: Cut-offs validated in one demographic (e.g., lean elective surgical patients) may not generalize to obese, emergency, or anatomically distorted cohorts. Surrogate outcomes: Evidence base dominated by laryngoscopic grade (CL ≥ 3), not patient-centered outcomes (intubation success, hypoxemia, complications, mortality)	Evidence quality: High-quality data limited by: (1) single-center observational studies with small samples; (2) methodological heterogeneity preventing definitive meta-analysis; (3) absence of multicenter RCTs demonstrating outcome benefit; (4) minimal validation outside elective adult populations [9,41,42,43,48]
Objective anatomical visualization	Provides real-time imaging of tongue, epiglottis, hyoid, laryngeal cartilages, vocal cords, and trachea with quantifiable measurements (DSE, HMDR, anterior neck soft-tissue thickness)	Moderate evidence: Reproducible in controlled settings with trained operators; inter-rater reliability varies (ICC 0.57–0.88) depending on parameter and operator experience. Image quality compromised in obesity, cervical pathology, or subcutaneous emphysema [9,44,49,50]
Diagnostic performance for difficult laryngoscopy	Meta-analyses report pooled sensitivity 76–86% and specificity 77–86% for predicting Cormack–Lehane grade ≥ 3, with AUROC 0.83–0.87 for DSE and anatomical position parameters—superior to individual clinical tests (Mallampati sensitivity ~38–52%) [8,9,41]	Critical caveats: (1) Most studies use laryngoscopic grade (surrogate outcome), not intubation success; (2) marked heterogeneity in cut-offs (DSE: 1.6–2.75 cm), scanning protocols, and patient populations limits generalizability; (3) small sample sizes (<200 patients in most trials); (4) limited validation in emergency/critically ill cohorts [9,34,42,43]

## Data Availability

This narrative review synthesizes previously published literature. No new datasets were generated. All cited studies are publicly accessible through their respective journal publications and are listed in the References section.

## Abbreviations

AI	Artificial Intelligence
ANS	Anterior Neck Soft-tissue Thickness
ASRA	American Society of Regional Anesthesia and Pain Medicine
AUC	Area Under the Curve
BMI	Body Mass Index
CICO	Cannot Intubate; Cannot Oxygenate
CTM	Cricothyroid Membrane
DSE	Distance from Skin to Epiglottis
DSHB	Distance Skin to Hyoid Bone
DSVC	Distance Skin to Vocal Cords
ED	Emergency Department
FONA	Front-of-Neck Access
HMD	Hyomental Distance
HMDR	Hyomental Distance Ratio
ICC	Intraclass Correlation Coefficient(s)
OR	Operating Room
OSA	Obstructive Sleep Apnea
POCUS	Point-of-Care Ultrasound
PES	Pre-Epiglottic Space
SANRA	Scale for the Assessment of Narrative Review Articles
US	Ultrasound
